# Expression Analysis of Genes Involved in Transport Processes in Mice with MPTP-Induced Model of Parkinson’s Disease

**DOI:** 10.3390/life12050751

**Published:** 2022-05-19

**Authors:** Margarita M. Rudenok, Maria I. Shadrina, Elena V. Filatova, Ivan N. Rybolovlev, Maxim S. Nesterov, Denis A. Abaimov, Ruslan A. Ageldinov, Anna A. Kolacheva, Michael V. Ugrumov, Petr A. Slominsky, Anelya Kh. Alieva

**Affiliations:** 1 Institute of Molecular Genetics of National Research Centre “Kurchatov Institute”, 2 Kurchatova Sq., 123182 Moscow, Russia; shadrina@img.ras.ru (M.I.S.); filatovaev@img.ras.ru (E.V.F.); i.rybolovlev@img.ras.ru (I.N.R.); slomin@img.ras.ru (P.A.S.); anelja.a@gmail.com (A.K.A.); 2 Scientific Center of Biomedical Technologies of the Federal Medical and Biological Agency of Russia, Svetlye Gory Village, Post Office Otradnoe, Krasnogorsk District, 143442 Moscow, Russia; mdulya@gmail.com (M.S.N.); ageldinov@gmail.com (R.A.A.); 3 The Research Centre of Neurology, 80, Volokolamskoye Shosse, 125367 Moscow, Russia; abaidenis@yandex.ru; 4 Koltsov Institute of Developmental Biology, Russian Academy of Sciences, 26 Vavilov Street, 119334 Moscow, Russia; annakolacheva@gmail.com (A.A.K.); michael.ugrumov@mail.ru (M.V.U.)

**Keywords:** Parkinson’s disease, neurological diseases, MPTP, neurodegeneration, molecular mechanisms, transport processes, gene expression, early diagnostics

## Abstract

Processes of intracellular and extracellular transport play one of the most important roles in the functioning of cells. Changes to transport mechanisms in a neuron can lead to the disruption of many cellular processes and even to cell death. It was shown that disruption of the processes of vesicular, axonal, and synaptic transport can lead to a number of diseases of the central nervous system, including Parkinson’s disease (PD). Here, we studied changes in the expression of genes whose protein products are involved in the transport processes (*Snca*, *Drd2*, *Rab5a*, *Anxa2*, and *Nsf*) in the brain tissues and peripheral blood of mice with MPTP (1-methyl-4-phenyl-1,2,3,6-tetrahydropyridine)-induced models of PD. We detected changes in the expressions of *Drd2*, *Anxa2*, and *Nsf* at the earliest modeling stages. Additionally, we have identified conspicuous changes in the expression level of *Anxa2* in the striatum and substantia nigra of mice with MPTP-induced models of PD in its early stages. These data clearly suggest the involvement of protein products in these genes in the earliest stages of the pathogenesis of PD.

## 1. Introduction

Among the most important processes in cell function are intracellular and extracellular transport [1,2,3]. These are carried out using transport proteins, cytoskeletal proteins, and transport vesicles [3,4].

Of particular importance is the cell transport in neurons. This is because of the structural features of these cell types, as in addition to the soma, they have very long processes (axons and dendrites) [3,5,6,7]. These require significant resources for the implementation of intracellular transport processes and provide one of the main functions of a neuron: transmission of a nerve impulse for synaptic transmission [8,9,10].

Nerve impulse transmission at the synapse is a complex physiological process that proceeds in several stages and includes multiple participants, such as synaptic vesicles, receptors, neurotransmitters, and transporters [11]. Vesicular transport is one of the most important components in synaptic transmission. In general, for a neuron, three main types of transport can be distinguished: axonal, vesicular, and synaptic [4,12]. Axonal transport ensures the integrity of the neuron and the connection of the soma with the synapse [12]. Vesicular transport is one of the most extensive types. Vesicles can be used for the transport of substances across the cell membrane by endocytosis and exocytosis. Numerous proteins and protein complexes participate in the formation, movement, and secretion of vesicle contents [9,10,13]. Synaptic transport is essential in the propagation of a nerve impulse and is also one of the most common modes of interaction between neurons [14,15,16].

Changes to transport mechanisms in a neuron can lead to the disruption of many cellular processes and even to cell death. Changes or disturbances in synaptic transmission can lead to a slowdown or interruption in the transmission of nerve impulses and to the disorganization of the brain and the organism as a whole [3,17].

Disruption of the processes of vesicular, axonal, and synaptic transport can lead to a number of diseases of the central nervous system [3,17,18,19,20,21,22,23,24,25,26,27,28], including Parkinson’s disease (PD) [4,29,30,31,32]. A key histopathology of PD is the presence of Lewy bodies (LBs) in the cell bodies and outgrowths of neurons in affected areas of the brain. They can have a toxic effect on the cell and isolate proteins vital for cell function [33]. There is evidence that the presence of LBs can be associated with disruptions to neuronal transport [28,34,35]. In addition, the presence of LBs complicates and changes transport in nerve cells [36,37,38]. This can lead to the accumulation of proteins in certain parts of the cell, obstruct synaptic signal transmission, and lead to slow neuronal death [39].

Previously, we performed a whole-transcriptome analysis of mouse brain tissues with MPTP (1-methyl-4-phenyl-1,2,3,6-tetrahydropyridine)-induced models of the early stages of PD [40,41]. It was shown that changes in the expression of proteins associated with transport could affect the very early stages of pathogenesis. In addition, the use of bioinformatic approaches made it possible to analyze the data and identify a number of genes influencing changes to the transport processes in the early stages of neurodegeneration.

Here, we present an account of the data obtained as a result of whole-transcriptome analysis and a study of the changes in expression of selected genes in MPTP-induced models of the early stages of PD.

## 2. Materials and Methods

### 2.1. Parkinson’s Disease Models

We used models of the presymptomatic stage of PD with decapitation of mice 6 h after last injection of MPTP (6 h-PSS), the presymptomatic stage of PD with decapitation of mice 24 h after last injection of MPTP (24 h-PSS), and the advanced presymptomatic (AdvPSS) and early symptomatic (ESS) stages of PD. Models were created as previously described [42,43]. Detailed information is presented in the work performed by our team earlier [44]. A brief description is provided in Table 1.

All efforts were made to minimize the potential suffering and discomfort of animals and care was taken according to the 3R rule (replacement, reduction, and refinement).

### 2.2. RNA Isolation and Expression Analysis of Individual Candidate Genes

RNA isolation from tissue samples from each animal as well as analysis of mRNA levels using reverse transcription and real-time PCR (TaqMan technology, StepOnePlus™ System, Applied Biosystems, USA) was carried out in accordance with the protocols described previously [44].

Primer and Probe design was produced using Beacon designer 7.0 software (v. 7.0, Premier Biosoft International, Palo Alto, CA, USA) and the nucleotide sequences of the *Snca*, *Drd2*, *Rab5a*, *Anxa2*, and *Nsf* genes and the reference genes *Bcat2* and *Psmd7* [45,46,47,48]. The sequences of gene-specific primers and probes are presented in Table 2.

### 2.3. Statistical Processing and Bioinformatic Analysis

The protocol of statistical and bioinformatic analysis is described in detail in the work carried out in our laboratory earlier [44].

## 3. Results

Previously, the whole-transcriptome analysis of the murine striatum and substantia nigra in models of the presymptomatic stage of PD with the decapitation of mice 6 h after the last injection of MPTP (6 h-PSS), the presymptomatic stage of PD with the decapitation of mice 24 h after the last injection of MPTP (24 h-PSS), and the advanced presymptomatic (AdvPSS) and early symptomatic (ESS) stages of PD was carried out in our laboratory. As a result, 170 differentially expressed genes that belonged to the “transport” clusters were identified [41].

**Figure 1:**
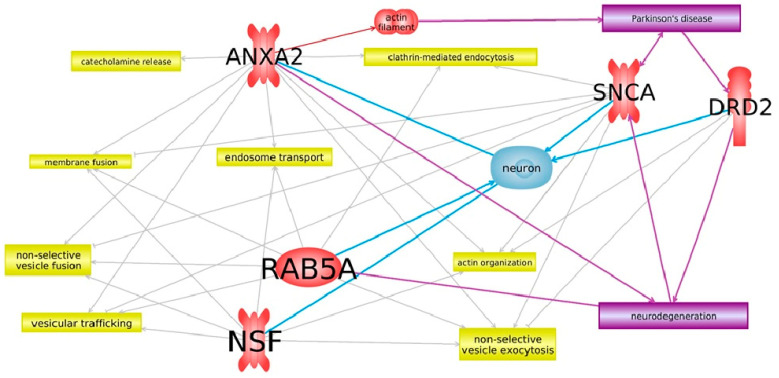
A network of genes that are somehow involved in the functioning of transport. Pathway Studio was used to build this network. Cellular processes are marked with a yellow box, genes and protein structures under study are marked with a red ellipse, disease (PD) and related processes are marked with a purple box, and a cell (neuron) is marked with light blue. Interactions between the gene and the object are indicated by arrows.

We performed a more detailed analysis of these genes using Pathway Studio^®^ software 12.0 (Elsevier, The Netherlands) [49,50]. A network of interactions was built using the keywords “Parkinson’s disease”, “transport”, “neuron”, and “neurodegeneration” under the condition of direct interactions between network participants. As a result, a network of interactions of five genes, *Snca*, *Drd2*, *Rab5a*, *Anxa2*, and *Nsf*, was built, corresponding to all keywords as closely as possible (Figure 1).

Further, an analysis of the expression levels of selected genes was performed at the early stages of the development of PD in the brain tissues and peripheral blood of mice using MPTP-induced models of the early stages of the disease [43]. Earlier, we obtained data on the expression of the *Snca* and *Drd2* genes in the brain tissues and peripheral blood using the AdvPSS and ESS models of PD [40]. This work presents data from previous [40] and current studies for building a holistic picture of the genes involved in this disease. The results of the expression analysis of the candidate genes are presented in Table 3.

The data presented in Table 3 demonstrate statistically significant changes in the expression of all the genes studied. However, the mRNA levels of *Drd2* in the peripheral blood of mice in the 6 h-PSS and 24 h-PSS models of PD were under the detection level of the methods used.

Fewer genes changed their expression levels in the tissues of animals with the 6 h-PSS and 24 h-PSS models than in those using the models of the more advanced stages of PD (12 versus 19).

At the earliest stage modeled (6 h-PSS), there were changes in gene expression only in the substantia nigra and peripheral blood, whereas there were increases in the relative mRNA levels for all genes; the relative expression levels of *Anxa2* and *Drd2* increased in the substantia nigra while *Snca*, *Rab5a*, and *Anxa2* transcript levels changed in the peripheral blood.

There was a change in the relative mRNA level of *Anxa2* in all studied tissues at 24 h after MPTP administration. Moreover, a large increase in mRNA level—almost 7.5-fold—was observed in the striatum. There was a significant decrease in the relative mRNA level of *Drd2* in the substantia nigra and an increase in the striatum by more than 1.5-fold. A change in expression at the mRNA level was also detected for *Nsf* in the striatum and peripheral blood.

There were significant changes in gene expression in the AdvPSS model of PD. There was an abrupt decrease in the relative mRNA level of *Anxa2* by 3.5-fold and a 2.5-fold increase for *Rab5a* in the substantia nigra. Decreased expression levels of *Rab5a* mRNA were also detected in the striatum and peripheral blood.

For the ESS model of PD, we found increases in the relative mRNA levels of *Nsf* and *Rab5a* in the substantia nigra. In addition, there was an abrupt 9-fold decrease for *Nsf* and an almost 2-fold increase for *Anxa2* in the striatum.

It is worth noting that the maximum number of significant data points on relative mRNA levels was obtained for *Drd2* and *Anxa2*, with 7 of 12 analyzed samples for each.

## 4. Discussion

Proteins encoded by the human genes of the monogenic forms of PD, *SNCA*, *VPS35*, *LRRK2*, and *ATP13A2* serve to maintain normal neuronal transport functions [36,51,52,53,54,55,56]. Mutations in these genes can contribute to the disruption of endo- and exocytosis and intracellular transport, which results in the incorrect redistribution of neurotransmitters, primarily dopamine (DA), in the synaptic gap and presynaptic terminals. These abnormalities can lead to impaired transport and neuronal death. However, despite the significance of these data [4], the entire spectrum of abnormal transport processes during the development of PD has not been fully described, and little is known about the roles of other genes involved in transport in the development of this disease.

The whole-transcriptome analysis of mouse brain tissues [40,41] and subsequent analysis of these data using Pathway Studio revealed potential significant participants in the transport processes in the early stages of the pathogenesis of PD (Figure 1). Therefore, the aim of this work was to study the expression levels of *Snca*, *Drd2*, *Rab5a*, *Nsf*, and *Anxa2* in the brain tissues and peripheral blood of mice containing MPTP-induced models of PD.

In the present study, we used MPTP-induced models of the presymptomatic stages of PD, 6 h-PSS and 24 h-PSS, and of the advanced presymptomatic (AdvPSS) and early symptomatic (ESS) stages of PD (with decapitation 2 weeks after MPTP administration) [42,43]. The administration of MPTP makes it possible to simulate the death of substantia nigra DAergic neurons, DA deficiency in the striatum, and the manifestation of the classic signs of PD [57,58]. PD models based on MPTP injections are considered the most adequate experimental models of this disease. An important advantage of using MPTP is that it selectively penetrates into DAergic neurons due to its high affinity for the DA transporter DAT and selectively inhibits complex I of the mitochondrial electron transport chain, leading to oxidative stress and impaired calcium homeostasis. These events lead to the degeneration of DAergic neurons through necrosis or apoptosis [59,60,61]. The introduction of MPTP makes it possible to simulate the various stages of neurodegeneration in PD, including the earliest ones. This makes it possible to study the specific patterns and mechanisms underlying neurodegeneration in PD at different stages of the development of the pathological process as well as study the compensatory mechanisms that occur in nerve cells. Today, MPTP is the gold standard for the study of Parkinson’s disease, including at the earliest stages [62].

Other types of models have a number of their own features. In addition to MPTP, one of the most common toxins is 6-hydroxydopamine (6-OHDA). The introduction of this toxin promotes the degeneration of catecholamine neurons, including DAergic neurons, since 6-OHDA has a high affinity for DA transporters. The neurotoxic effect of 6-OHDA is mediated by a two-stage mechanism: at the first stage, the toxin accumulates in catecholamic neurons; at the second stage, a change in cellular homeostasis and neuron death occurs. Because of its similarity to endogenous catecholamines, 6-OHDA is taken up by the DAT and NAT transporters. The oxidation of 6-OHDA by monoamine oxidase (MAO-A) promotes the formation of hydrogen peroxide (H_2_O_2_), which is not only cytotoxic but also causes the formation of oxygen radicals [63]. An increase in the concentration of reactive oxygen species and other reactive substances caused by the entry of 6-OHDA into the cell leads to a rapid depletion of intracellular antioxidants, which, in turn, enhances neurotoxicity, leading to cell death [64].

A significant disadvantage of 6-OHDA is its inability to penetrate the BBB, which leads to its direct administration by stereotaxic method into the striatum and/or substantia nigra and less often into other parts of the brain [65,66,67,68]. Moreover, when using 6-OHDA, it is rather problematic to model the presymptomatic stages of PD since the very nature of toxin administration causes the rapid death of DA neurons [65,69].

Genetic models can mainly show only that variant of the pathogenesis of PD caused by a specific genetic defect. In addition, genetic models do not clearly reproduce the behavioral and neurodegenerative changes characteristic of PD. In addition, familial forms of PD account for only 5–15% of all cases of the disease, so this type of model cannot cover the entire spectrum of the causes of PD. Another disadvantage of such models is that they practically do not reproduce the presymptomatic stages of PD [70,71,72,73,74,75].

There are also models employing the use of amphetamine psychostimulants and reserpine. Currently, they are used very rarely to model the presymptomatic stages of PD due to their high nonspecificity. The principle of action is very different from the compounds described above and is associated with the release of DA from the terminals of neurons into the cytosol, leading to a lack of DA in the striatum [76,77,78,79]. These models do not show clear symptoms of parkinsonism and can be used to study the dysfunction of the catecholaminergic system as a whole.

In this work, the tissues of the striatum, substantia nigra, and peripheral blood of mice with MPTP-induced PD models were used. The striatum and substantia nigra are mainly subject to neurodegeneration in PD in the earliest stages. Blood was examined to further explore candidate genes as potential transcriptional peripheral biomarkers for this disease.

Table 3 demonstrates a greater number of significant changes in the tissues of the brain and peripheral blood of mice with models of AdvPSS and ESS of PD compared with those with 6 h-PSS and 24 h-PSS models. We observed more pronounced changes at the transcriptome level at the advanced stages of the development of the pathological process in PD. These results correlate with our previous data that showed that the number of differentially expressed genes increased with the progression of the disease [41,44].

In our previous study, we obtained data on the expression of *Snca* and *Drd2* in the brain tissues and peripheral blood of animals using AdvPSS and ESS models [40] (Table 3). Mutations in the human *DRD2* gene encoding the DA uptake receptor and release inhibitor D2 might be associated with some psychiatric and neurological diseases [80]. Alpha-synuclein (SNCA), encoded by the *SNCA* gene, is a presynaptic protein and is actively involved in the functioning of various types of neurons [81]. It is worth noting that *SNCA* can be involved in the development of both familial and sporadic forms of PD [82]. Alieva et al. (2017) demonstrated that these genes changed their expression levels in the brain tissues of mice with MPTP-induced models of AdvPSS and ESS, and the most pronounced and significant changes were observed in the substantia nigra [40]. These data indicated the involvement of the *Snca* and *Drd2* genes in the neurodegenerative processes of PD. At the same time, an analysis of earlier time points (6 h-PSS and 24 h-PSS models of PD) suggested that changes in the *Snca* mRNA levels did not occur at the early stages of neurodegeneration. Here, we observed an increase in the relative level of *Drd2* mRNA in the striatum and a decrease in the substantia nigra 24 h after the last injection of MPTP. Thus, this gene might be involved in pathogenesis at the very early stages of PD.

There were significant changes in the expression of *Nsf* mRNA in the striatum and substantia nigra in the PD models studied. *Nsf*, encoding the N-ethylmaleimide-sensitive factor, is required at all stages of the endosome pathway, from the endoplasmic reticulum to the Golgi apparatus and from the Golgi apparatus to the plasma membrane [83]. Several studies have demonstrated that the dysfunction of this protein can lead to the disruption of intracellular transport, which includes not only a decrease in vesicular traffic but also damage to the cytoskeleton [84,85,86]. An increase in the expression of *Nsf* mRNA in the substantia nigra in the ESS model of PD indicates that, at this stage of neurodegeneration, the mechanisms of intracellular transport are enhanced precisely in the nerve cell body. However, there are insufficient resources to support these processes in nerve cell bodies and neuronal outgrowths. This is reflected in a decrease in the *Nsf* mRNA levels in the striatum at the same simulated stage.

*Rab5a*, encoded by a member of the RAS oncogene family, as well as Nsf, is required for the fusion of endosomes with the plasma membrane [87,88]. This protein is involved in the conversion of early endosomes to late ones [89]. In 2004, Delprato et al. showed that motor neurons degenerate during Rab5a dysfunction [90]. The *Rab5a* gene showed increased expression in the substantia nigra in the AdvPSS and ESS models of PD. Moreover, the mRNA level of this gene decreased only in the striatum in the AdvPSS model but increased in the control samples in the ESS model. It is likely that an increase in the mRNA level of this gene at these stages of neurodegeneration in the substantia nigra might lead to the development of the compensatory mechanisms observed in the striatum in the ESS model.

In the present study, *Anxa2* showed the maximum number of significant results at the mRNA level (Table 3). The protein annexin A2 encoded by this gene performs a number of biological functions such as the regulation of the organization of organelle membranes [91], participation in clathrin-mediated endocytosis, and other types of vesicular transport [92]. Annexin A2 is also involved in the formation of the actin cytoskeleton [93]. Babbin et al. (2007) showed that inhibition of *Anxa2* expression in cell cultures can lead to the destabilization of actin filaments and their subsequent depolymerization [94].

Although reports on the dysfunction of annexin A2 appear primarily in the field of cancer research, a role for this protein in diseases of the nervous system, such as Alzheimer’s disease [95,96] and Niemann–Pick type C disease, has also been demonstrated [97]. However, there is very little evidence that the *Anxa2* gene is directly involved in the pathogenesis of PD. It was shown that astrocytes expressing pathogenic Lrrk2 showed a significant decrease in the expression of Anxa2 at the protein level and a subsequent decrease in intracellular α-synuclein [98].

**Figure 2:**
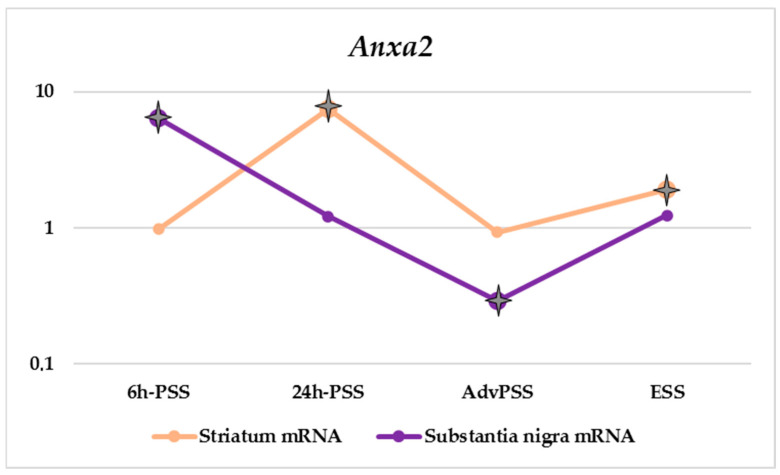
Relative mRNA and protein levels of *Anxa2* in striatum and substantia nigra of mice with MPTP-induced models of PD. The expression level studied in the control was taken as 1. 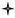 *p*-Value ≤ 0.05.

To maintain proper synaptic functioning, a neuron needs to concentrate all its resources precisely in the near-synaptic region, including the enhanced expression of proteins involved in vesicular transport. Our data support this assumption (Table 3 and Figure 2). We observed a significant increase in the mRNA level of the *Anxa2* gene in the substantia nigra at the earliest modeled stage (6 h-PSS). In this case, it is likely that the cells were trying to transport RNA to the neuronal terminal, which is reflected in the striatum in the 24 h-PSS model of PD. This indicates that neurons are trying to compensate for the lack of resources caused by the development of neurodegeneration.

The study of gene expression in the blood opens up broad prospects in the search for genes that change expression at different stages of the disease, which will make it possible to better understand the mechanisms of PD pathogenesis. Blood cells, in particular lymphocytes, have some characteristics typical of a dopaminergic neuron: they contain both enzymes for dopamine synthesis, a dopamine membrane transporter, and a number of dopamine receptors are expressed. It has been assumed that the changes found in the peripheral blood of patients with PD might reflect the processes occurring in the DAergic neurons of the substantia nigra [99,100,101] and that blood can be taken for analysis to further study candidate genes as transcriptional peripheral markers of this disease. In our work, we also analyzed the changes in gene expression in the peripheral blood of mice with MPTP-induced PD. Previously, a correlation of changes in the relative levels of the *Snca* and *Drd2* mRNAs was revealed in the substantia nigra and peripheral blood [42]. Our work also revealed statistically significant changes in the peripheral blood for all the studied genes. At the same time, one can see a trend toward a decrease in the mRNA levels in the models of the more advanced stages of PD. Such changes may reflect an increase in the neurodegenerative process through a decrease in the expression of genes involved in cell transport processes.

## 5. Conclusions

Here, we studied changes in the expression of genes whose protein products are involved in transport processes (*Snca*, *Drd2*, *Rab5a*, *Anxa2*, and *Nsf*) in the brain tissues and peripheral blood of mice with MPTP-induced models of PD. We detected changes in the expressions of *Drd2*, *Anxa2*, and *Nsf* at the earliest modeling stages. These findings suggest that changes in transport already occur in the early stages of neurodegeneration.

Moreover, we are the first to report conspicuous changes in the expression levels of *Anxa2* in the striatum and substantia nigra of mice with MPTP-induced models of the early stages of PD. These data clearly suggest the involvement of this gene in the compensatory mechanisms in the early stages of the pathogenesis of PD.

## Figures and Tables

**Table 1:** Description of used models.

	6 h-PSS	24 h-PSS	AdvPSS	ESS
Dose of MPTP	12 mg/kg	12 mg/kg	12 mg/kg	12 mg/kg
Number and frequency of injections	4 injections at 2-h intervals	4 injections at 2-h intervals	2 injections at 2-h intervals	4 injections at 2-h intervals
Withdrawal time after the last injection of MPTP	6 h	24 h	2 weeks	2 weeks
Change in DA levels in the striatum	89% reduction	72% increase	56% reduction	75% reduction
Death of DAergic neurons in substantia nigra	33% reduction	unchanged	26% reduction	43% reduction
Changes in animal behavior	unchanged	unchanged	unchanged	a decrease in motor activity was detected

6 h-PSS—model of the presymptomatic stage of PD with decapitation of mice 6 h after last injection of MPTP; 24 h-PSS—model of the presymptomatic stage of PD with decapitation of mice 24 h after last injection of MPTP; AdvPSS—model of the advanced presymptomatic stage of PD; ESS—early symptomatic stage of PD. MPTP—1-methyl-4-phenyl-1,2,3,6-tetrahydropyridine.

**Table 2:** Nucleotide sequences of gene-specific primers and probes.

Gene	Nucleotide Sequence
*Snca* *(Synuclein Alpha)* NM_001042451.2 *	Probe: 5′-VIC-CCTCCTCACCCTTGCCCATCTGGTCC-BHQ2-3′ Forward primer: 5′-AGTGGAGGGAGCTGGGAATATAG-3′ Reverse primer: 5′-GCATGTCTTCCAGGATTCCTTCC-3′
*Drd2* *(Dopamine Receptor D2)* NM_010077.2 *	Probe: 5′-ROX-CAGCCAGCAGATGATGAACACACCAAGA-BHQ2-3′ Forward primer: 5′-TCCCAGCAGAAGGAGAAGAAAG-3′ Reverse primer: 5′-TGTATATTCAGGATGTGCGTGATG-3′
*Rab5a* *(RAB5A*, *Member RAS Oncogene Family)* NM_025887.4 *	Probe: 5′-VIC-CATCTGCATAGGACTGTGCTTCC-BHQ2-3′ Forward primer: 5′-GCAAGTCCTAATATTGTGATA-3′ Reverse primer: 5′-CTCCATAAATAATAAGCTGTTG-3′
*Anxa2* *(Annexin A2)* NM_007585.3 *	Probe: 5′-VIC-AGAAGGACATCATCTCTGACACATCTG-BHQ2-3′ Forward primer: 5′-GAGTGTACAAGGAAATGTAC-3′ Reverse primer: 5′-CGTAGTCAATAACTGAGC-3′
*Nsf* *(N-ethylmaleimide sensitive fusion protein)* NM_008740.4 *	Probe: 5′-VIC-CGTCCTCACCATCACATGCTG-BHQ2-3′ Forward primer: 5′-CCCTACTGATGAATTATCTTTA-3′ Reverse primer: 5′-TCCTCAGCGTAAATATGTA-3′
*Bcat2* *(Branched chain aminotransferase 2)* NM_001243053.1 *	Probe: 5′-FAM-CGGATACACTCCAACAGCTCCTGCTTGT-BHQ1-3′ Forward primer: 5′-TCAACATGGACAGGATGCTACG-3′ Reverse primer: 5′-CCAGTCTTTGTCTACTTCAATGAGC-3′
*Psmd7* *(Proteasome 26S Subunit*, *Non-ATPase 7)* NM_010817.2 *	Probe: 5′-FAM-AGTCCTAGGTCCTTTGGCTTCACGTCGA-BHQ1-3′ Forward primer: 5′-CTGCACAAGAATGATATCGCCATC-3′ Reverse primer: 5′-CTCCACTGAGATGTAGGCTTCG-3′

* Numbers in the database GenBank (Accession numbers). FAM, ROX and VIC—fluorescent dyes; BHQ1 and BHQ2—fluorescence quenchers.

**Table 3:** Relative mRNA levels of the genes studied in brain and peripheral blood of mice with MPTP-induced models of early stages of PD.

Genes	Striatum				Substantia Nigra				Peripheral Blood			
	6 h-PSS (*n* = 10)	24 h-PSS (*n* = 10)	Adv-PSS (*n* = 10)	ESS (*n* = 10)	6 h-PSS (*n* = 10)	24 h-PSS (*n* = 10)	Adv-PSS (*n* = 10)	ESS (*n* = 10)	6 h-PSS (*n* = 10)	24 h-PSS (*n* = 10)	Adv-PSS (*n* = 10)	ESS (*n* = 10)
*Drd2*	0.94 ^1^	**1.76**	0.90 *	0.42 *	**1.34**	**0.57**	3.55 *	0.27 *	**—**	—	0.90 *	0.38 *
	0.77–1.18 ^2^	**1.25–2.39**	0.17–1.09	**0.33–0.86**	**1.24–1.46**	**0.46–0.81**	**3.14–5.80**	**0.13–0.59**			0.75–1.52	**0.27–0.44**
*Snca*	1.10	0.87	0.93 *	0.73 *	0.87	0.49	7.52 *	0.17 *	**3.37**	0.74	0.69 *	0.20 *
	1.01–1.33	0.78–1.41	0.12–1.00	0.61–1.30	0.81–1.13	0.33–1.06	**6.42–8.36**	**0.16–0.36**	**2.12–4.29**	0.60–0.93	0.21–1.82	**0.14–0.36**
*Rab5a*	1.03	1.01	**0.57**	1.12	0.93	1.01	**2.68**	**2.43**	**1.81**	0.61	**0.36**	0.96
	0.83–1.52	0.82–1.11	**0.47–0.85**	0.39–1.59	0.72–1.08	0.88–1.44	**1.79–4.89**	**2.01–3.38**	**1.56–2.72**	0.39–1.30	**0.22–0.47**	0.73–1.82
*Anxa2*	0.98	**7.41**	0.93	**1.92**	**6.38**	**1.21**	**0.29**	1.24	**2.88**	**0.71**	1.30	1.05
	0.84–1.22	**5.80–9.60**	0.68–2.20	**1.37–2.75**	**5.38–6.47**	**0.72–3.83**	**0.19–1.01**	0.99–1.56	**1.12–5.11**	**0.36–0.87**	0.83–1.96	0.73–1.70
*Nsf*	0.98	**1.29**	**2.69**	**0.11**	1.13	1.03	1.32	**3.50**	1.14	**0.58**	**0.72**	1.07
	0.88–1.09	**1.00–1.59**	**1.67–3.27**	**0.07–0.33**	1.12–1.25	0.82–1.31	0.71–3.13	**2.47–5.21**	1.06–3.18	**0.39–1.67**	**0.46–0.84**	0.79–1.13

^1^ Median. ^2^ 25–75—quartiles. *n* = 10 for each group. The levels of the mRNA of the genes studied in the control group were taken as 1. The data with *p* < 0.05 and fold change ≥1.5 are bold and highlighted. The data with *p* < 0.05 and fold change ˂1.5 are bold but not highlighted. Dashes indicate samples in which analysis was not performed. * The results obtained in previous studies [40]. 6 h-PSS—model of the presymptomatic stage of PD with decapitation of mice 6 h after last injection of MPTP; 24 h-PSS—model of the presymptomatic stage of PD with decapitation of mice 24 h after last injection of MPTP; AdvPSS—model of the advanced presymptomatic stage of PD; ESS—early symptomatic stage of PD.

## Data Availability

Not applicable.

## References

[1] Wilson D.B. (1978). Cellular transport mechanisms. Annu. Rev. Biochem..

[2] Berger F., Keller C., Muller M.J., Klumpp S., Lipowsky R. (2011). Co-operative transport by molecular motors. Biochem. Soc. Trans..

[3] Benarroch E.E. (2012). Membrane trafficking and transport: Overview and neurologic implications. Neurology.

[4] Abeliovich A., Gitler A.D. (2016). Defects in trafficking bridge Parkinson’s disease pathology and genetics. Nature.

[5] Goldstein L.S., Yang Z. (2000). Microtubule-based transport systems in neurons: The roles of kinesins and dyneins. Annu. Rev. Neurosci..

[6] Maeder C.I., Shen K., Hoogenraad C.C. (2014). Axon and dendritic trafficking. Curr. Opin. Neurobiol..

[7] Bridgman P.C. (2004). Myosin-dependent transport in neurons. J. Neurobiol..

[8] Isola A.L., Chen S. (2017). Exosomes: The Messengers of Health and Disease. Curr. Neuropharmacol..

[9] Rizo J., Sudhof T.C. (2012). The membrane fusion enigma: SNAREs, Sec1/Munc18 proteins, and their accomplices—Guilty as charged?. Annu. Rev. Cell Dev. Biol..

[10] Kononenko N.L., Haucke V. (2015). Molecular mechanisms of presynaptic membrane retrieval and synaptic vesicle reformation. Neuron.

[11] Gondre-Lewis M.C., Park J.J., Loh Y.P. (2012). Cellular mechanisms for the biogenesis and transport of synaptic and dense-core vesicles. Int. Rev. Cell Mol. Biol..

[12] Grafstein B., Forman D.S. (1980). Intracellular transport in neurons. Physiol. Rev..

[13] Hannah M.J., Schmidt A.A., Huttner W.B. (1999). Synaptic vesicle biogenesis. Annu. Rev. Cell Dev. Biol..

[14] Eccles J.C., Kramer K., Krayer O., Lehnartz E., Muralt A.V., Weber H.H. (1961). The Mechanism of Synaptic Transmission. Ergebnisse der Physiologie Biologischen Chemie und Experimentellen Pharmakologie.

[15] Huettner J.E. (2003). Kainate receptors and synaptic transmission. Prog. Neurobiol..

[16] Katz B., Miledi R. (1967). A study of synaptic transmission in the absence of nerve impulses. J. Physiol..

[17] Bronfman F.C., Escudero C.A., Weis J., Kruttgen A. (2007). Endosomal transport of neurotrophins: Roles in signaling and neurodegenerative diseases. Dev. Neurobiol..

[18] Halim N.D., Weickert C.S., McClintock B.W., Hyde T.M., Weinberger D.R., Kleinman J.E., Lipska B.K. (2003). Presynaptic proteins in the prefrontal cortex of patients with schizophrenia and rats with abnormal prefrontal development. Mol. Psychiatry.

[19] Gabriel S.M., Davidson M., Haroutunian V., Powchik P., Bierer L.M., Purohit D.P., Perl D.P., Davis K.L. (1996). Neuropeptide deficits in schizophrenia vs. Alzheimer’s disease cerebral cortex. Biol. Psychiatry.

[20] Shankar G.M., Walsh D.M. (2009). Alzheimer′s disease: Synaptic dysfunction and Abeta. Mol. Neurodegener..

[21] Waites C.L., Garner C.C. (2011). Presynaptic function in health and disease. Trends Neurosci..

[22] Willis M., Leitner I., Jellinger K.A., Marksteiner J. (2011). Chromogranin peptides in brain diseases. J. Neural Transm..

[23] Lauterborn J.C., Rex C.S., Kramar E., Chen L.Y., Pandyarajan V., Lynch G., Gall C.M. (2007). Brain-derived neurotrophic factor rescues synaptic plasticity in a mouse model of fragile X syndrome. J. Neurosci. Off. J. Soc. Neurosci..

[24] Annangudi S.P., Luszpak A.E., Kim S.H., Ren S., Hatcher N.G., Weiler I.J., Thornley K.T., Kile B.M., Wightman R.M., Greenough W.T. (2010). Neuropeptide Release is Impaired in a Mouse Model of Fragile X Mental Retardation Syndrome. ACS Chem. Neurosci..

[25] Verhoeven K., De Jonghe P., Coen K., Verpoorten N., Auer-Grumbach M., Kwon J.M., FitzPatrick D., Schmedding E., De Vriendt E., Jacobs A. (2003). Mutations in the small GTP-ase late endosomal protein RAB7 cause Charcot-Marie-Tooth type 2B neuropathy. Am. J. Hum. Genet..

[26] Henderson L.P., Lin L., Prasad A., Paul C.A., Chang T.Y., Maue R.A. (2000). Embryonic striatal neurons from niemann-pick type C mice exhibit defects in cholesterol metabolism and neurotrophin responsiveness. J. Biol. Chem..

[27] Chevalier-Larsen E., Holzbaur E.L. (2006). Axonal transport and neurodegenerative disease. Biochim. Biophys. Acta.

[28] Volpicelli-Daley L.A. (2017). Effects of alpha-synuclein on axonal transport. Neurobiol. Dis..

[29] Bossers K., Meerhoff G., Balesar R., Van Dongen J.W., Kruse C.G., Swaab D.F., Verhaagen J. (2009). Analysis of Gene Expression in Parkinson’s Disease: Possible Involvement of Neurotrophic Support and Axon Guidance in Dopaminergic Cell Death. Brain Pathol..

[30] Hauser M.A., Li Y.-J., Xu H., Noureddine M.A., Shao Y.S., Gullans S.R., Scherzer C.R., Jensen R.V., McLaurin A.C., Gibson J.R. (2005). Expression Profiling of Substantia Nigra in Parkinson Disease, Progressive Supranuclear Palsy, and Frontotemporal Dementia With Parkinsonism. Arch Neurol..

[31] Miller R.M., Kiser G.L., Kaysser-Kranich T.M., Lockner R.J., Palaniappan C., Federoff H.J. (2006). Robust dysregulation of gene expression in substantia nigra and striatum in Parkinson’s disease. Neurobiol. Dis..

[32] Bieri G., Gitler A.D., Brahic M. (2018). Internalization, axonal transport and release of fibrillar forms of alpha-synuclein. Neurobiol. Dis..

[33] Kalia L.V., Lang A.E. (2015). Parkinson’s disease. Lancet.

[34] Yamamoto S., Fukae J., Mori H., Mizuno Y., Hattori N. (2006). Positive immunoreactivity for vesicular monoamine transporter 2 in Lewy bodies and Lewy neurites in substantia nigra. Neurosci. Lett..

[35] Power J.H., Barnes O.L., Chegini F. (2017). Lewy Bodies and the Mechanisms of Neuronal Cell Death in Parkinson’s Disease and Dementia with Lewy Bodies. Brain Pathol..

[36] Lin M.K., Farrer M.J. (2014). Genetics and genomics of Parkinson’s disease. Genome Med..

[37] Tsika E., Glauser L., Moser R., Fiser A., Daniel G., Sheerin U.M., Lees A., Troncoso J.C., Lewis P.A., Bandopadhyay R. (2014). Parkinson’s disease-linked mutations in VPS35 induce dopaminergic neurodegeneration. Hum. Mol. Genet..

[38] Alter S.P., Lenzi G.M., Bernstein A.I., Miller G.W. (2013). Vesicular integrity in Parkinson’s disease. Curr. Neurol. Neurosci. Rep..

[39] Mukherjee A., Biswas A., Das S.K. (2016). Gut dysfunction in Parkinson’s disease. World J. Gastroenterol..

[40] Alieva A.K., Filatova E.V., Kolacheva A.A., Rudenok M.M., Slominsky P.A., Ugrumov M.V., Shadrina M.I. (2017). Transcriptome Profile Changes in Mice with MPTP-Induced Early Stages of Parkinson’s Disease. Mol. Neurobiol..

[41] Alieva A.K., Zyrin V.S., Rudenok M.M., Kolacheva A.A., Shulskaya M.V., Ugryumov M.V., Slominsky P.A., Shadrina M.I. (2018). Whole-Transcriptome Analysis of Mouse Models with MPTP-Induced Early Stages of Parkinson’s Disease Reveals Stage-Specific Response of Transcriptome and a Possible Role of Myelin-Linked Genes in Neurodegeneration. Mol. Neurobiol..

[42] Ugrumov M.V., Khaindrava V.G., Kozina E.A., Kucheryanu V.G., Bocharov E.V., Kryzhanovsky G.N., Kudrin V.S., Narkevich V.B., Klodt P.M., Rayevsky K.S. (2011). Modeling of presymptomatic and symptomatic stages of parkinsonism in mice. Neuroscience.

[43] Kolacheva A.A., Kozina E.A., Volina E.V., Ugryumov M.V. (2014). Time course of degeneration of dopaminergic neurons and respective compensatory processes in the nigrostriatal system in mice. Dokl. Biol.Sci. Proc. Acad. Sci. USSR Biol. Sci. Sect. Transl. Russ..

[44] Rudenok M.M., Alieva A.K., Starovatykh J.S., Nesterov M.S., Stanishevskaya V.A., Kolacheva A.A., Ugryumov M.V., Slominsky P.A., Shadrina M.I. (2020). Expression analysis of genes involved in mitochondrial biogenesis in mice with MPTP-induced model of Parkinson’s disease. Mol. Genet. Metab. Rep..

[45] Chang Y.C., Ding Y., Dong L., Zhu L.J., Jensen R.V., Hsiao L.L. (2018). Differential expression patterns of housekeeping genes increase diagnostic and prognostic value in lung cancer. PeerJ.

[46] Haslinger D., Waltes R., Yousaf A., Lindlar S., Schneider I., Lim C.K., Tsai M.M., Garvalov B.K., Acker-Palmer A., Krezdorn N. (2018). Loss of the Chr16p11.2 ASD candidate gene QPRT leads to aberrant neuronal differentiation in the SH-SY5Y neuronal cell model. Mol. Autism.

[47] Hoerndli F.J., Toigo M., Schild A., Gotz J., Day P.J. (2004). Reference genes identified in SH-SY5Y cells using custom-made gene arrays with validation by quantitative polymerase chain reaction. Anal. Biochem..

[48] Warrington J.A., Nair A., Mahadevappa M., Tsyganskaya M. (2000). Comparison of human adult and fetal expression and identification of 535 housekeeping/maintenance genes. Physiol. Genom..

[49] Hettne K.M., Thompson M., van Haagen H.H., van der Horst E., Kaliyaperumal R., Mina E., Tatum Z., Laros J.F., van Mulligen E.M., Schuemie M. (2016). The Implicitome: A Resource for Rationalizing Gene-Disease Associations. PLoS ONE.

[50] Zoetmulder M., Biernat H.B., Nikolic M., Korbo L., Friberg L., Jennum P.J. (2014). Prepulse inhibition is associated with attention, processing speed, and 123I-FP-CIT SPECT in Parkinson’s disease. J. Parkinson’s Dis..

[51] Brunger A.T. (2000). Structural insights into the molecular mechanism of Ca(2+)-dependent exocytosis. Curr. Opin. Neurobiol..

[52] Steger M., Tonelli F., Ito G., Davies P., Trost M., Vetter M., Wachter S., Lorentzen E., Duddy G., Wilson S. (2016). Phosphoproteomics reveals that Parkinson′s disease kinase LRRK2 regulates a subset of Rab GTPases. eLife.

[53] Inoshita T., Arano T., Hosaka Y., Meng H., Umezaki Y., Kosugi S., Morimoto T., Koike M., Chang H.Y., Imai Y. (2017). Vps35 in cooperation with LRRK2 regulates synaptic vesicle endocytosis through the endosomal pathway in Drosophila. Hum. Mol. Genet..

[54] Esteves A.R., Swerdlow R.H., Cardoso S.M. (2014). LRRK2, a puzzling protein: Insights into Parkinson′s disease pathogenesis. Exp. Neurol..

[55] Demirsoy S., Martin S., Motamedi S., van Veen S., Holemans T., Van den Haute C., Jordanova A., Baekelandt V., Vangheluwe P., Agostinis P. (2017). ATP13A2/PARK9 regulates endo-/lysosomal cargo sorting and proteostasis through a novel PI(3, 5)P2-mediated scaffolding function. Hum. Mol. Genet..

[56] Park J.S., Blair N.F., Sue C.M. (2015). The role of ATP13A2 in Parkinson’s disease: Clinical phenotypes and molecular mechanisms. Mov. Disord..

[57] German D.C., Nelson E.L., Liang C.L., Speciale S.G., Sinton C.M., Sonsalla P.K. (1996). The neurotoxin MPTP causes degeneration of specific nucleus A8, A9 and A10 dopaminergic neurons in the mouse. Neurodegeneration.

[58] Meredith G.E., Totterdell S., Potashkin J.A., Surmeier D.J. (2008). Modeling PD pathogenesis in mice: Advantages of a chronic MPTP protocol. Parkinsonism. Relat. Disord..

[59] Gerlach M., Riederer P. (1996). Animal models of Parkinson′s disease: An empirical comparison with the phenomenology of the disease in man. J. Neural. Transm..

[60] Langston J.W., Ballard P., Tetrud J.W., Irwin I. (1983). Chronic Parkinsonism in humans due to a product of meperidine-analog synthesis. Science.

[61] Przedborski S., Jackson-Lewis V., Djaldetti R., Liberatore G., Vila M., Vukosavic S., Almer G. (2000). The parkinsonian toxin MPTP: Action and mechanism. Restor. Neurol. Neurosci..

[62] Salari S., Bagheri M. (2019). In vivo, in vitro and pharmacologic models of Parkinson’s disease. Physiol. Res..

[63] Cohen G., Heikkila R.E., Allis B., Cabbat F., Dembiec D., MacNamee D., Mytilineou C., Winston B. (1976). Destruction of sympathetic nerve terminals by 6-hydroxydopamine: Protection by 1-phenyl-3-(2-thiazolyl)-2-thiourea, diethyldithiocarbamate, methimazole, cysteamine, ethanol and n-butanol. J. Pharmacol. Exp. Ther..

[64] Blum D., Torch S., Lambeng N., Nissou M., Benabid A.L., Sadoul R., Verna J.M. (2001). Molecular pathways involved in the neurotoxicity of 6-OHDA, dopamine and MPTP: Contribution to the apoptotic theory in Parkinson′s disease. Prog. Neurobiol..

[65] Deumens R., Blokland A., Prickaerts J. (2002). Modeling Parkinson’s Disease in Rats: An Evaluation of 6-OHDA Lesions of the Nigrostriatal Pathway. Exp. Neurol..

[66] Lorigados P.L., Fuentes N.P., Alvarez G.L., McRae A., Serrano S.T., Blanco L.L., Macías G.R. (2002). Nerve growth factor levels in Parkinson disease and experimental parkinsonian rats. Brain Res..

[67] Dimatelis J.J., Hendricks S., Hsieh J., Vlok N.M., Bugarith K., Daniels W.M., Russell V.A. (2013). Exercise partly reverses the effect of maternal separation on hippocampal proteins in 6-hydroxydopamine-lesioned rat brain. Exp. Physiol..

[68] Sun W., Sugiyama K., Asakawa T., Yamaguchi H., Akamine S., Ouchi Y., Magata Y., Namba H. (2011). Dynamic changes of striatal dopamine D2 receptor binding at later stages after unilateral lesions of the medial forebrain bundle in Parkinsonian rat models. Neurosci. Lett..

[69] Carman L.S., Gage F.H., Shults C.W. (1991). Partial lesion of the substantia nigra relation between extent of lesion. Brain Res..

[70] Gonzalez S., Mena M.A., Lastres-Becker I., Serrano A., de Yebenes J.G., Ramos J.A., Fernandez-Ruiz J. (2005). Cannabinoid CB(1) receptors in the basal ganglia and motor response to activation or blockade of these receptors in parkin-null mice. Brain Res..

[71] Rial D., Castro A.A., Machado N., Garcao P., Goncalves F.Q., Silva H.B., Tome A.R., Kofalvi A., Corti O., Raisman-Vozari R. (2014). Behavioral phenotyping of Parkin-deficient mice: Looking for early preclinical features of Parkinson’s disease. PLoS ONE.

[72] Kelm-Nelson C.A., Yang K.M., Ciucci M.R. (2015). Exercise Effects on Early Vocal Ultrasonic Communication Dysfunction in a PINK1 Knockout Model of Parkinson’s Disease. J. Parkinson’s Dis..

[73] Bishop M.W., Chakraborty S., Matthews G.A., Dougalis A., Wood N.W., Festenstein R., Ungless M.A. (2010). Hyperexcitable substantia nigra dopamine neurons in PINK1- and HtrA2/Omi-deficient mice. J. Neurophysiol..

[74] Rousseaux M.W., Qu D., Hewitt S.J., Seang S., Kim R.H., Slack R.S., Schlossmacher M.G., Lagace D.C., Mak T.W., Park D.S. (2012). Progressive dopaminergic cell loss with unilateral-to-bilateral progression in a genetic model of Parkinson disease. Proc. Natl. Acad. Sci. USA.

[75] Yue Z., Lachenmayer M.L. (2011). Genetic LRRK2 models of Parkinson’s disease: Dissecting the pathogenic pathway and exploring clinical applications. Mov. Disord. Off. J. Mov. Disord. Soc..

[76] Bernheimer H., Birkmayer W., Hornykiewicz O., Jellinger K., Seitelberger F. (1973). Brain dopamine and the syndromes of Parkinson and Huntington. Clinical, morphological and neurochemical correlations. J. Neurol. Sci..

[77] Betarbet R., Sherer T.B., Greenamyre J.T. (2002). Animal models of Parkinson’s disease. BioEssays News Rev.Mol. Cell. Dev. Biol..

[78] Fibiger H.C., Mogeer E.G. (1971). Effect of acute and chronic methamphetamine treatment on tyrosine hydroxylase activity in brain and adrenal medulla. Eur. J. Pharmacol..

[79] Sonsalla P.K., Nicklas W.J., Heikkila R.E. (1989). Role for excitatory amino acids in methamphetamine-induced nigrostriatal dopaminergic toxicity. Science.

[80] Noble E.P. (2003). D2 dopamine receptor gene in psychiatric and neurologic disorders and its phenotypes. Am. J. Med. Genet. Part B Neuropsychiatr.Genet. Off. Publ. Int. Soc. Psychiatr. Genet..

[81] Burre J., Sharma M., Tsetsenis T., Buchman V., Etherton M.R., Sudhof T.C. (2010). Alpha-synuclein promotes SNARE-complex assembly in vivo and in vitro. Science.

[82] Ozansoy M., Basak A.N. (2013). The central theme of Parkinson’s disease: Alpha-synuclein. Mol. Neurobiol..

[83] Whiteheart S.W., Schraw T., Matveeva E.A. (2001). N-ethylmaleimide sensitive factor (NSF) structure and function. Int. Rev. Cytol..

[84] Hayashi M., Taniguchi S., Ishizuka Y., Kim H.S., Wataya Y., Yamamoto A., Moriyama Y. (2001). A homologue of N-ethylmaleimide-sensitive factor in the malaria parasite Plasmodium falciparum is exported and localized in vesicular structures in the cytoplasm of infected erythrocytes in the brefeldin A-sensitive pathway. J. Biol. Chem..

[85] Nunes P., Haines N., Kuppuswamy V., Fleet D.J., Stewart B.A. (2006). Synaptic vesicle mobility and presynaptic F-actin are disrupted in a N-ethylmaleimide-sensitive factor allele of Drosophila. Mol. Biol. Cell.

[86] Faugaret D., Chouinard F.C., Harbour D., El Azreq M.-A., Bourgoin S.G. (2011). An essential role for phospholipase D in the recruitment of vesicle amine transport protein-1 to membranes in human neutrophils. Biochem. Pharm..

[87] Woodman P. (1998). Vesicle transport: More work for the Rabs?. Curr. Biol..

[88] Tanmay B., Kumar R.J. (2014). Rab proteins: The key regulators of intracellular vesicle transport. Exp. Cell Res..

[89] Roth B.L., Willins D.L., Kroeze W.K. (1998). G protein-coupled receptor (GPCR) trafficking in the central nervous system: Relevance for drugs of abuse. Drug Alcohol. Depend..

[90] Delprato A., Merithew E., Lambright D.G. (2004). Structure, exchange determinants, and family-wide rab specificity of the tandem helical bundle and Vps9 domains of Rabex-5. Cell.

[91] Luo M., Hajjar K.A. (2013). Annexin A2 system in human biology: Cell surface and beyond. Semin. Thromb. Hemost..

[92] Bharadwaj A., Bydoun M., Holloway R., Waisman D. (2013). Annexin A2 heterotetramer: Structure and function. Int. J. Mol. Sci..

[93] Filipenko N.R., Waisman D.M. (2001). The C Terminus of Annexin II Mediates Binding to F-actin. J. Biol. Chem..

[94] Babbin B.A., Parkos C.A., Mandell K.J., Winfree L.M., Laur O., Ivanov A.I., Nusrat A. (2007). Annexin 2 regulates intestinal epithelial cell spreading and wound closure through Rho-related signaling. Am. J. Pathol..

[95] Joanna B.-P. (2004). Annexins in the Central Nervous System: Are they Neuroprotective or Proapoptotic Agents?. Med. Chem. Rev. Online.

[96] Brandt R., Bakota L. (2017). Microtubule dynamics and the neurodegenerative triad of Alzheimer′s disease: The hidden connection. J. Neurochem..

[97] Domon M.M., Besson F., Bandorowicz-Pikula J., Pikula S. (2011). Annexin A6 is recruited into lipid rafts of Niemann-Pick type C disease fibroblasts in a Ca^2+^-dependent manner. Biochem. Biophys. Res. Commun..

[98] Streubel-Gallasch L., Giusti V., Sandre M., Tessari I., Plotegher N., Giusto E., Masato A., Iovino L., Battisti I., Arrigoni G. (2021). Parkinson’s Disease-Associated LRRK2 Interferes with Astrocyte-Mediated Alpha-Synuclein Clearance. Mol. Neurobiol..

[99] Barbanti P., Fabbrini G., Ricci A., Cerbo R., Bronzetti E., Caronti B., Calderaro C., Felici L., Stocchi F., Meco G. (1999). Increased expression of dopamine receptors on lymphocytes in Parkinson’s disease. Mov. Disord..

[100] Buttarelli F.R., Capriotti G., Pellicano C., Prosperi D., Circella A., Festa A., Giovannelli M., Tofani A., Pontieri F.E., Scopinaro F. (2009). Central and peripheral dopamine transporter reduction in Parkinson′s disease. Neurol. Res..

[101] Nagai Y., Ueno S., Saeki Y., Soga F., Hirano M., Yanagihara T. (1996). Decrease of the D3 dopamine receptor mRNA expression in lymphocytes from patients with Parkinson’s disease. Neurology.

